# Fewer COVID‐19 Neurological Complications with Dexamethasone and Remdesivir

**DOI:** 10.1002/ana.26536

**Published:** 2022-11-09

**Authors:** Alexander Grundmann, Chieh‐Hsi Wu, Marc Hardwick, J. Kenneth Baillie, Peter J M Openshaw, Malcolm G. Semple, Dankmar Böhning, Sarah Pett, Benedict D. Michael, Rhys H. Thomas, Ian Galea, for the ISARIC4C investigators

**Affiliations:** ^1^ Clinical Neurosciences, Clinical and Experimental Sciences, Faculty of Medicine University of Southampton Southampton UK; ^2^ Department of Neurology Wessex Neurological Centre, University Hospital Southampton NHS Foundation Trust Southampton UK; ^3^ Statistics, Mathematical Sciences, and Faculty of Social Sciences University of Southampton Southampton UK; ^4^ Roslin Institute University of Edinburgh, Easter Bush Edinburgh UK; ^5^ Intensive Care Unit Royal Infirmary of Edinburgh Edinburgh UK; ^6^ National Heart and Lung Institute Imperial College London London UK; ^7^ Imperial College Healthcare NHS Trust London UK; ^8^ NIHR Health Protection Research Unit for Emerging and Zoonotic Infections Institute of Infection, Veterinary and Ecological Sciences, University of Liverpool Liverpool UK; ^9^ Department of Respiratory Medicine Alder Hey Children's Hospital Liverpool UK; ^10^ Medical Research Council Clinical Trials Unit Institute of Clinical Trials and Methodology, University College London London UK; ^11^ Institute for Global Health University College London London UK; ^12^ Department of Clinical Infection Microbiology and Immunology Institute of Infection, Veterinary, and Ecological Sciences, University of Liverpool Liverpool UK; ^13^ Department of Neurology The Walton Centre NHS Foundation Trust Liverpool UK; ^14^ Translational and Clinical Research Institute University of Newcastle Newcastle upon Tyne UK; ^15^ Department of Neurology Royal Victoria Infirmary Newcastle upon Tyne UK

## Abstract

**Objective:**

The objective of this study was to assess the impact of treatment with dexamethasone, remdesivir or both on neurological complications in acute coronavirus diease 2019 (COVID‐19).

**Methods:**

We used observational data from the International Severe Acute and emerging Respiratory Infection Consortium World Health Organization (WHO) Clinical Characterization Protocol, United Kingdom. Hospital inpatients aged ≥18 years with laboratory‐confirmed severe acute respiratory syndrome‐coronavirus 2 (SARS‐CoV‐2) infection admitted between January 31, 2020, and June 29, 2021, were included. Treatment allocation was non‐blinded and performed by reporting clinicians. A propensity scoring methodology was used to minimize confounding. Treatment with remdesivir, dexamethasone, or both was assessed against the standard of care. The primary outcome was a neurological complication occurring at the point of death, discharge, or resolution of the COVID‐19 clinical episode.

**Results:**

Out of 89,297 hospital inpatients, 64,088 had severe COVID‐19 and 25,209 had non‐hypoxic COVID‐19. Neurological complications developed in 4.8% and 4.5%, respectively. In both groups, neurological complications were associated with increased mortality, intensive care unit (ICU) admission, worse self‐care on discharge, and time to recovery. In patients with severe COVID‐19, treatment with dexamethasone (n = 21,129), remdesivir (n = 1,428), and both combined (n = 10,846) were associated with a lower frequency of neurological complications: OR = 0.76 (95% confidence interval [CI] = 0.69–0.83), OR = 0.69 (95% CI = 0.51–0.90), and OR = 0.54 (95% CI = 0.47–0.61), respectively. In patients with non‐hypoxic COVID‐19, dexamethasone (n = 2,580) was associated with less neurological complications (OR = 0.78, 95% CI = 0.62–0.97), whereas the dexamethasone/remdesivir combination (n = 460) showed a similar trend (OR = 0.63, 95% CI = 0.31–1.15).

**Interpretation:**

Treatment with dexamethasone, remdesivir, or both in patients hospitalized with COVID‐19 was associated with a lower frequency of neurological complications in an additive manner, such that the greatest benefit was observed in patients who received both drugs together. ANN NEUROL 2023;93:88–102

Coronavirus disease 2019 (COVID‐19) is associated with a range of multisystem complications, causing significant morbidity which may overshadow the acute illness,^1^ especially in younger and less clinically vulnerable patients. Although existing therapeutics have been shown to reduce the severity and mortality of acute COVID‐19, their effects on functional outcomes, including a return to the premorbid level of functioning, are less well‐defined.^2^, ^3^, ^4^ Of all systemic complications following COVID‐19, neurological syndromes are associated with the worst functional outcomes,^1^ and include stroke, encephalopathies, neuropsychiatric, and inflammatory syndromes.^5^, ^6^, ^7^, ^8^ Even in non‐hospitalized patients, COVID‐19 is associated with longitudinal changes in brain structure and cognition.^9^ As COVID‐19 is projected to become an endemic seasonal infection,^10^, ^11^ the incidence, severity, and demographics of neurological complications may translate into a significant socioeconomic burden of care, in terms of physical and cognitive disability, across multiple decades. Evidence‐based treatment strategies aiming to reduce the morbidity arising from neurological complications of COVID‐19 are therefore of urgent public health importance.

The disease processes leading to neurological complications include viral‐driven endotheliitis, systemic inflammation leading to coagulopathy, cytokine toxicity, blood brain barrier disruption, antibody and cell‐mediated autoimmunity, as well as the consequences of severe prolonged illness.^7^, ^12^, ^13^ Neurological complications increase with the severity of COVID‐19,^7^ which is in turn associated with a higher viral load in the upper respiratory tract.^14^ Uncontrolled systemic viremia can lead to a hyper‐inflammatory immune response, which may facilitate bystander neurological autoimmunity.^15^, ^16^ It follows that existing treatments licensed for COVID‐19 may prevent or lessen the impact of neurological complications through reducing viral replication and the severity of inflammation, although this has not been investigated.

Remdesivir is a repurposed antiviral medication which has shown broad antiviral action in vitro against emerging coronaviruses, including Middle‐East Respiratory Syndrome coronavirus (MERS‐CoV), and severe acute respiratory syndrome‐coronavirus 1 and 2 (SARS‐CoV‐1 and SARS‐CoV‐2).^17^ In the ACTT‐1 trial, remdesivir demonstrated a significantly greater chance of clinical improvement in adults hospitalized with COVID‐19 and requiring supplemental oxygen.^18^ Whereas the SOLIDARITY trial found no significant effect for remdesivir on time to recovery, patients in the remdesivir group were admitted for a longer period to receive their treatment, potentially confounding this result.^4^ More recently, in the PINETREE study,^19^ remdesivir markedly reduced COVID‐19‐related hospitalization in patients at high risk of disease progression. Finally, remdesivir was shown to reduce the time to clinical improvement in a separate retrospective analysis of hospitalized patients with COVID‐19.^20^ Taken together, these results suggest that remdesivir has potential to prevent neurological complications by reducing the severity of COVID‐19, likely through inhibiting viral replication.

Dexamethasone is now a widely used treatment for COVID‐19, after the RECOVERY trial reported a reduction in 28‐day mortality in hospitalized patients with COVID‐19 receiving either invasive ventilation or supplemental oxygen.^2^ Rather than affecting viral replication, it is thought to work by mitigating hyper‐inflammatory organ injury, which may in turn prevent neurological complications.

This study therefore aimed to establish whether remdesivir, dexamethasone, or both, in addition to the standard of care, was associated with a reduction in the frequency of major neurological complications in adults admitted to the hospital with COVID‐19.

## Methods

### Study Design and Participants

We analyzed COVID‐19 neurological complications and remdesivir and dexamethasone use in adults admitted to the hospital with COVID‐19 using data from the International Severe Acute and emerging Respiratory Infection Consortium (ISARIC) World Health Organization (WHO) Clinical Characterization Protocol UK (CCP‐UK; study registration ISRCTN66726260). This is an ongoing prospective noninterventional study using case report forms (CRFs) developed for “disease X,” long before the emergence of SARS‐CoV‐2. This cohort now comprises data from over 303,000 hospital inpatients with COVID‐19 from 306 sites across the United Kingdom, but at the time of data lock 184,986 eligible cases were available for analysis. The data are open for access at https://isaric4c.net/sample_access/. The ISARIC's Coronavirus Clinical Characterization Consortium (ISARIC4C) collected these data for urgent public health‐related purposes, including assessing the clinical characteristics, ethnicity, risk factors, and complications experienced by people admitted to the hospital with COVID‐19^1^, ^21^, ^22^ and the impact of medications on patient outcomes.^23^, ^24^ Materials relating to CCP‐UK, including protocol, revision history, CRFs, and recruiting sites, are available online.^21^, ^25^ The ISARIC WHO CCP‐UK received ethical approval from the South Central Oxford C Research Ethics Committee in England (13/SC/0149), the Scotland A Research Ethics Committee in Scotland (20/SS/0028), and the WHO Ethics Review Committee (RPC571 and RPC572, April 25, 2013). All outcomes were recorded at the point of death, discharge, or 28 days’ admission, whichever happened first.

A detailed protocol including eligibility criteria and analytic plan was defined a priori.^26^ Predefined inclusion criteria included age of 18 years and older, and admission to the hospital between January 31, 2020, and June 29, 2021, with laboratory confirmed SARS‐CoV‐2 infection. Exclusion criteria included COVID‐19 vaccination and nosocomial COVID‐19. The study was performed and reported in accordance with STROBE guidelines.^27^

### Definitions

Patients requiring supplemental oxygen at any point during their admission (most often for O_2_ saturations <94%) were defined as having severe COVID‐19, whereas those not requiring supplemental oxygen at any point during their admission were defined as having non‐hypoxic COVID‐19. Major neurological complications were only considered if occurring de novo, after hospital admission with COVID‐19. The neurological diagnosis was determined by the physicians looking after the patient, and included any one of the following: stroke, seizure, meningitis/encephalitis (likely para‐infectious rather than infective), or any other neurological complication. The ISARIC CCP UK CRF version 10.1 (REDCap, Vanderbilt University and REDCap consortium) is pragmatic^25^ so the category “other neurological complication” captures rare complications, such as central nervous system vasculitis, acute disseminated encephalomyelitis, posterior reversible encephalopathy syndrome, and Guillain‐Barre syndrome.^28^

Intensive care unit (ICU) admission was defined as admission for intensive or high dependency care at any point during admission. Worse self‐care was defined as either self‐care being recorded as worse relative to the pre‐admission baseline, or a need for continued hospitalization, at the resolution of the COVID‐19 clinical episode. Time to recovery was defined as the time in days from admission to discharge or resolution of the COVID‐19‐related clinical episode.

#### Covariates

Nine baseline covariates were used to derive the propensity score in all treatment groups. These included sex at birth, age, self‐reported ethnicity, Dalhousie University Clinical Frailty score,^29^ and smoking status (current, former, or never). Self‐reported ethnicity was transcribed from the health care record, with patients categorized into East Asian, South Asian, West Asian, Black, White, Latin American, Aboriginal/First Nations, and Other ethnic minority. For the purposes of this analysis, the collective ethnic minorities were collapsed into a single group. Time in days from first symptom onset to hospital admission, severity of pulmonary infection (defined by requirement for noninvasive or invasive ventilation at any point during admission), and contraindications for remdesivir (only in those treatment groups prescribed remdesivir) were also included. Finally, a combined score of clinically significant comorbidities was used, ranging from 0 to 14, as defined in the pre‐published protocol.^26^ The comorbidities were cardiac disease, inclusive of congenital heart disease, not including hypertension; hypertension; any chronic pulmonary disease, including asthma; chronic kidney disease; any rheumatologic disorder; any moderate or severe liver disease; any chronic hematological disease; any chronic neurological disorder including dementia; type 1 or 2 diabetes; any malignant neoplasm; patients on immunosuppression therapies; obesity; solid organ transplant recipients; patients with rare diseases, and inborn errors of metabolism that significantly increase the risk of infections, such as severe combined immunodeficiency. Further detail on all the covariates and the calculation of the combined comorbidities score is available in the published protocol.^26^

### Treatments

This was a prospective observational study so patients and clinicians were not blinded to treatment allocation. Data were collected by independent research nurses and clinical trainees not involved in treatment allocation or clinical management. The research team was independent to patient care and only handled anonymized data. All treatment courses were prescribed locally by treating clinicians according to national guidelines, which varied during the study period.^26^ In all cases, nonexposure was defined as the standard of care, defined as the best available treatment without a remdesivir or dexamethasone prescription at any point.

#### Remdesivir

The UK guidelines recommended prescription initially as part of the Early Access to Medicines Scheme (EAMS) for people aged 12 years and older affected with severe COVID‐19. Following July 2020, the EAMS scheme was withdrawn and a prescription was recommended in patients newly hospitalized with COVID‐19, within 10 days of symptom onset and only in nonventilated patients. Remdesivir was contraindicated in patients with an estimated glomerular filtration rate (eGFR) <30 ml/min/1.73m^2^ and those known to be pregnant.^30^

#### Dexamethasone

Dexamethasone was commissioned for use in COVID‐19 in the United Kingdom on June 16, 2020, in patients requiring supplemental oxygen to meet prescribed oxygen saturation levels.^31^

#### Treatment Groups

Because patients were treated with either or both treatments of interest, in addition to the standard of care, the following treatment group comparisons were performed: (1) remdesivir alone versus the standard of care, (2) dexamethasone alone versus the standard of care, and (3) combined remdesivir and dexamethasone versus the standard of care.

Treatment exposure with remdesivir was considered as either starting on the day of admission or at any point during admission. Analyses were performed separately for patients with severe and non‐hypoxic COVID‐19.

### Statistical Methods

All analyses were conducted as prespecified in the protocol published a priori.^26^ A complete case analysis was conducted; patients missing data in the CRFs for the admission day or discharge were excluded, as were those with missing data for neurological complications. The duration of case observation encompassed the day of admission until either the point of discharge from the hospital, death, or continued hospitalization without ongoing care needs related to COVID‐19. To adjust for confounding between treatment groups, a propensity score methodology^32^ was used. A matching approach was not used to enable the entire study population to be used, thus maximizing the sample size, and instead the propensity score was used to adjust for confounding. The propensity score for each treatment comparison was determined by a logistic regression, using the covariates detailed above, and the treatment comparison pair of interest as the outcome variable. Each propensity score was subsequently used as a covariate to separately adjust all final logistic or linear regression analyses of outcome on treatment. For comparison between patients with and without neurological complications, a logistic or linear regression was performed, including the variables used in the propensity score as covariates. Statistical analyses were performed independently, and results reproduced, by Alexander Grundmann and Chieh‐Hsi Wu using RStudio version 4.0.5 (RStudio, Boston, MA, USA).^33^ Propensity scores were calculated using the “MatchIt” package.^34^

## Results

### Cohort Demographics and Clinical Characteristics in Severe COVID‐19

**FIGURE 1:**
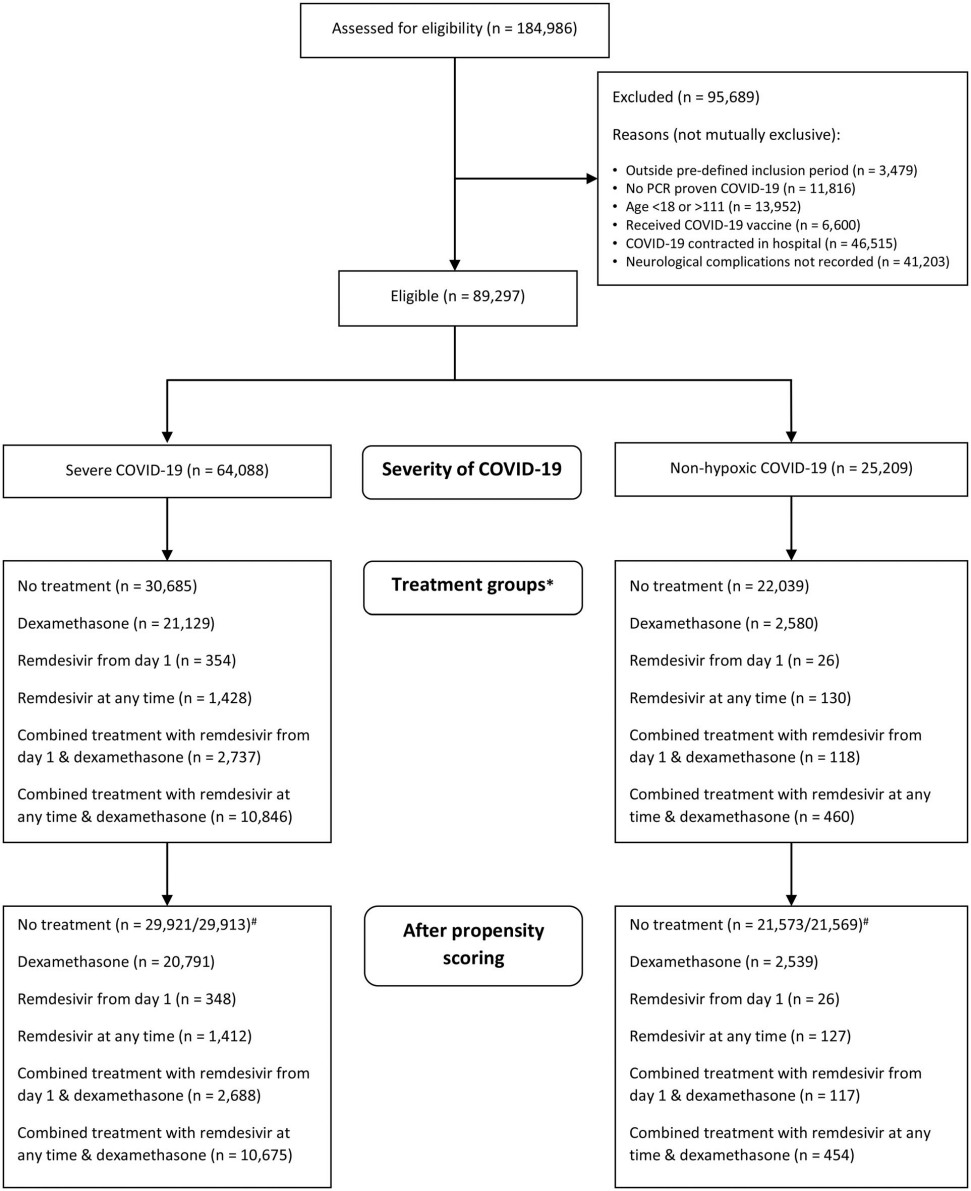
Consolidated Standards of Reporting Trials (CONSORT) flowchart showing the number of patients meeting eligibility criteria, reasons for exclusion from the study, the 2 groups of severe and non‐hypoxic COVID‐19, and relevant treatment groups. * Treatment groups are not mutually exclusive. Patients receiving remdesivir from day 1 are represented in both remdesivir from day 1 and remdesivir at any time treatment groups.^#^ Two control groups are present because the propensity score was calculated twice, one with and one without contraindications to remdesivir as a covariate; because the latter is less restrictive, the sample size is slightly larger. COVID‐19 = coronavirus disease 2019.

Of 184,986 patients assessed for eligibility, 89,297 were included in the study and 64,088 had severe COVID‐19. Reasons for exclusion are outlined in Figure 1. Demographic and clinical characteristics are presented in Tables 1 and 2 (with further details in Table S1). In brief, for patients with severe COVID‐19, the median age was 70 years (interquartile range [IQR] = 57–81 years), 59% of patients were men and 71% were identified as having White ethnicity. Neurological complications occurred in 4.9% (n = 3,104) of the cohort and consisted of seizures (n = 692), meningitis/encephalitis (n = 142), stroke (n = 923), and other complications (n = 1,642).

**TABLE 1:** Demographic and Clinical Characteristics of the Study Population with Severe and Non‐Hypoxic COVID‐19 (See Tables S1 and S3 in Supporting Information for More Details)

Characteristic	Severe COVID‐19	Non‐hypoxic COVID‐19
Sample size	n = 64,088	n = 25,209
Age, median (IQR)	70 (56–81)	72 (54–83)
Sex, n (%)		
F	26,460 (41)	12,667 (50)
M	37,544 (59)	12,508 (50)
Not specified	68 (0.1)	30 (0.1)
Ethnicity, n (%)		
BAME	6,787 (11)	2,606 (10)
White	45,499 (72)	18,094 (72)
Other	3,799 (6.0)	1,327 (5.3)
Not specified	7,491 (12)	2,961 (12)
Comorbidities score, median (IQR)	2.00 (1.00–3.00)	2.00 (1.00–3.00)
Clinical Frailty Score, median (IQR)	4.00 (2.00–6.00)	4.00 (3.00–6.00)
Smoking status, n (%)		
Current smoker	2,851 (4.5)	1,491 (6.0)
Former smoker	14,931 (23)	4,532 (18)
Never smoked	18,782 (30)	7,336 (29)
Not specified	26,996 (42)	11,642 (47)
Ventilated, n (%)	19,204 (30)	0 (0)
Time from symptom onset to admission, days, median (IQR)	5.0 (2.0–9.0)	3 (0–7)
Patients with neurological complication, n (%)	3,104 (4.8)	1,141 (4.5)
Mortality, n (%)	20,234 (32)	2,307 (9.2)
ICU admission, n (%)	13,629 (21)	235 (0.9)
Worse self‐care at discharge, n (%)	7,778 (19)	2,706 (13)
Time to recovery, days, median (IQR)	8 (5–14)	5 (2–10)

BAME = Black, Asian, and minority ethnic; COVID‐19 = coronavirus disease 2019; ICU = intensive care unit; IQR = interquartile range.

**TABLE 2:** Demographic and Clinical Characteristics of Patients with Severe COVID‐19, Organized by Treatment Group (See Tables S1 and S3 in Supporting Information for More Details)

Characteristic	No treatment	Dexamethasone	Remdesivir at any time	Remdesivir from day 1	Combined treatment with remdesivir at any time	Combined treatment with remdesivir from day 1
	n = 30,685	n = 21,129	n = 1,428	n = 354	n = 10,846	n = 2,737
Age, median (IQR)	74 (60–84)	68 (55–79)	63 (53–74)	60 (51–71)	62 (52–74)	60 (50–70)
Sex, n (%)						
F	13,180 (43)	8,662 (41)	535 (37)	126 (36)	4,083 (38)	975 (36)
M	17,465 (57)	12,435 (59)	891 (62)	228 (64)	6,753 (62)	1,761 (64)
Not specified	30 (<0.1)	28 (0.1)	2 (0.1)	0 (0)	8 (<0.1)	1 (<0.1)
Ethnicity, n (%)						
BAME	2,923 (9.6)	2,263 (11)	204 (14)	49 (14)	1,397 (13)	377 (14)
White	22,335 (73)	15,148 (72)	923 (65)	213 (61)	7,093 (66)	1,674 (62)
Other	1,681 (5.5)	1,259 (6.0)	89 (6.3)	24 (6.9)	770 (7.2)	207 (7.6)
Not specified	3,549 (12)	2,266 (11)	197 (14)	64 (18)	1,479 (14)	451 (17)
Comorbidities score, median (IQR)	2.00 (1.00–3.00)	2.00 (1.00–3.00)	2.00 (1.00–3.00)	2.00 (1.00–3.00)	2.00 (1.00–3.00)	1.00 (0.00–2.00)
Clinical Frailty Score, median (IQR)	5.00 (3.00–7.00)	3.00 (2.00–5.00)	3.00 (2.00–5.00)	2.00 (2.00–4.00)	3.00 (2.00–4.00)	2.00 (2.00–3.00)
Smoking status, n (%)						
Current smoker	1,474 (4.8)	874 (4.2)	55 (3.9)	9 (2.6)	448 (4.2)	101 (3.8)
Former smoker	6,834 (22)	5,221 (25)	316 (22)	62 (18)	2,560 (24)	588 (22)
Never smoked	8,080 (26)	6,390 (31)	413 (29)	118 (34)	3,899 (36)	1,026 (38)
Not specified	14,159 (46)	8,391 (40)	635 (45)	162 (46)	3,811 (36)	976 (36)
Ventilated, n (%)	7,541 (25)	6,137 (29)	760 (53)	198 (56)	4,766 (44)	1,215 (44)
Time from symptom onset to admission, days, median (IQR)	4.0 (1.0–8.0)	6.0 (2.0–9.0)	6.0 (3.0–8.0)	7.0 (4.0–9.0)	6.0 (3.0–9.0)	7.0 (4.0–9.0)
Patients with neurological complication, n (%)	1,967 (6.4)	780 (3.7)	56 (3.9)	18 (5.1)	301 (2.8)	63 (2.3)
Mortality, n (%)	11,490 (37)	5,919 (28)	472 (33)	95 (27)	2,353 (22)	448 (16)
ICU admission, n (%)	5,330 (17)	4,379 (21)	614 (43)	180 (51)	3,306 (31)	887 (32)
Worse self‐care at discharge, n (%)	3,590 (21)	2,582 (18)	220 (25)	56 (24)	1,386 (17)	343 (16)
Time to recovery, days, median (IQR)	9 (5–16)	7 (4–12)	10 (6–16)	9 (5–15)	8 (6–13)	7 (5–11)

All percentages are that of the group represented in the column. Some patients had more than one complication so the sum of individual diagnoses is greater than the number of patients with neurological complications.

BAME = Black, Asian, and minority ethnic; COVID‐19 = coronavirus disease 2019; ICU = intensive care unit; IQR = interquartile range.

### Association of Mortality and Morbidity with Treatments in Severe COVID‐19

To validate the dataset and ensure conformity with published data,^24^, ^35^, ^36^ we first studied the effects of treatment groups on mortality in severe COVID‐19 (Table 3). Treatment with dexamethasone was associated with reduced mortality (OR = 0.86, 95% CI = 0.83–0.90), whereas treatment with remdesivir was not (OR = 0.97, 95% CI = 0.87–1.09). Combined treatment with remdesivir and dexamethasone was associated with a further reduction in mortality (OR = 0.67, 95% CI = 0.63–0.71), which was likely synergistic because log(0.67) < [log(0.86) + log(0.97)]. Treatment with dexamethasone was associated with a reduction in ICU admission (OR = 0.92, 95% CI = 0.88–0.97), whereas treatment with remdesivir was associated with increased ICU admission (OR = 1.58, 95% CI = 1.36–1.85). None of the treatment groups were associated with worse self‐care at discharge or an increased time to recovery.

**TABLE 3:** Mortality and Morbidity (Admission to Intensive or High‐Dependency Care, Ability for Self‐Care at Discharge, and Length of Inpatient Stay) in Patients with Severe COVID‐19 According to the Treatment Group

Treatment group	Mortality	95% CI	ICU admission	95% CI	Self‐care worse	95% CI	Change in time to recovery (days)	95% CI
No treatment n = 30,685	1		1		1		0	
Dexamethasone n = 21,129	0.86	0.83, 0.90	0.92	0.88, 0.97	0.91	0.86, 0.96	−2.41	−3.45, −1.36
Remdesivir from day 1 n = 354	0.81	0.63, 1.03	2.00	1.51, 2.63	1.10	0.8, 1.49	−0.89	−2.71, 0.93
Remdesivir at any time n = 1,428	0.97	0.87, 1.09	1.58	1.36, 1.85	1.11	0.95, 1.30	0.02	−0.94, 0.97
Combined treatment with remdesivir from day 1 n = 2,737	0.53	0.47, 0.59	0.99	0.90, 1.10	0.73	0.64, 0.83	−2.92	−3.54, −2.30
Combined treatment with remdesivir at any time n = 10,846	0.67	0.63, 0.71	0.96	0.90, 1.02	0.79	0.73, 0.85	−1.21	−1.60, −0.82

All values reported are odds ratios adjusted by the relevant propensity score, apart from change in time to recovery (days) which is a linear regression adjusted by the relevant propensity score.

COVID‐19 = coronavirus disease 2019; CI = confidence interval; ICU = intensive care unit.

### Effects of Neurological Complications in Severe COVID‐19

Patients with severe COVID‐19 and neurological complications (n = 3,104), compared to severe COVID‐19 without neurological complications (n = 60,984; Table S2), had an increased mortality (OR 1.36, 95% CI 1.25–1.47), ICU admission (OR 1.54, 95% CI 1.41–1.6), likelihood of worse self‐care on discharge (OR 3.79, 95% CI 3.37–4.26) and an increased time to recovery (9.65 days, 95% CI 7.12–12.17 days).

### Association of Neurological Complications with Treatments in Severe COVID‐19

**FIGURE 2:**
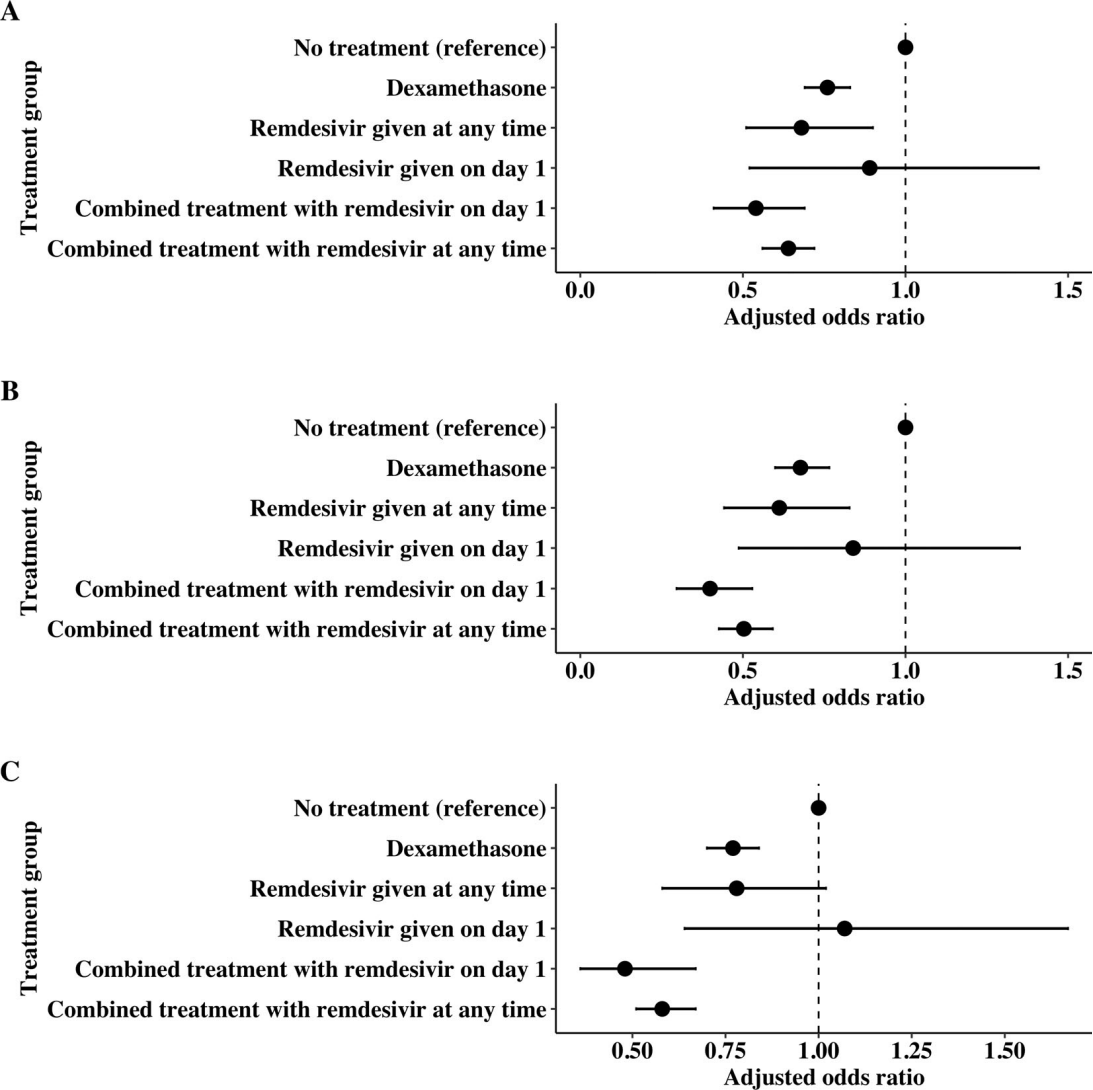
Odds ratios showing the likelihood of neurological complications in patients with severe COVID‐19 by treatment group. All odds ratios are adjusted by the relevant propensity score for each treatment group. Whiskers represent 95% confidence intervals. (A) Protocolled analysis. (B) Sensitivity analysis excluding cases prior to the approval of remdesivir and dexamethasone in the United Kingdom, and including the time since the start of the pandemic as a covariate in the generation of the propensity score. (C) Sensitivity analysis using the whole dataset, not adjusting for ventilation status in the propensity score. COVID‐19 = coronavirus disease 2019.

Treatment with dexamethasone (n = 21,129) was associated with a lower frequency of neurological complications (OR = 0.76, 95% CI = 0.69–0.83), as was treatment with remdesivir at any time (n = 1,428, OR = 0.69, 95% CI = 0.51–0.90;) (Fig 2A). Combined treatment (n = 10,846) was associated with a larger reduction (OR = 0.54, 95% CI = 0.47–0.61), in keeping with an additive effect since log(0.54) ≈ [log(0.76) + log(0.69)]. An exploratory subgroup analysis was performed within the 4 individual neurological complication categories (stroke, epilepsy, meningitis/encephalitis, and other neurological complications), with similar results; some analyses were limited by a low sample size (Table 4). Treatment with remdesivir, dexamethasone, or the combination was associated with a lower frequency of stroke, seizure, and meningitis in most instances but not with other neurological complications (Table 4), likely due to the heterogenous nature of this group.

**TABLE 4:** Subgroup Sensitivity Analysis Examining the Association Between Treatments and Frequency of Neurological Complications within the Four Diagnostic Subgroups

	Severe COVID‐19			Non‐hypoxic COVID‐19		
	OR	CI low	CI high	OR	CI low	CI high
**Seizure**						
Dexamethasone	0.41	0.33	0.52	0.70	0.40	1.12
Remdesivir any time	0.28	0.10	0.61	0.86	0.05	3.89
Remdesivir from day 1	NA	NA	NA	NA	NA	NA
Combined treatment with remdesivir at any time	0.37	0.27	0.51	0.51	0.08	1.61
Combined treatment with remdesivir from day 1	0.32	0.16	0.57	NA	NA	NA
**Stroke**						
Dexamethasone	0.57	0.48	0.68	0.47	0.29	0.72
Remdesivir any time	0.53	0.29	0.88	1.05	0.17	1.32
Remdesivir from day 1	0.66	0.20	1.57	NA	NA	NA
Combined treatment with remdesivir at any time	0.47	0.37	0.60	0.50	0.12	1.32
Combined treatment with remdesivir from day 1	0.40	0.24	0.64	0.71	0.04	3.19
**Meningitis/encephalitis**						
Dexamethasone	0.42	0.25	0.67	1.08	0.37	2.47
Remdesivir any time	0.23	0.01	1.06	NA	NA	NA
Remdesivir from day 1	NA	NA	NA	NA	NA	NA
Combined treatment with remdesivir at any time	0.20	0.08	0.43	1.20	0.07	5.57
Combined treatment with remdesivir from day 1	0.13	0.01	0.61	NA	NA	NA
**Other neurological complication**						
Dexamethasone	0.98	0.88	1.10	0.89	0.68	1.14
Remdesivir any time	0.85	0.59	1.17	1.26	0.39	2.99
Remdesivir from day 1	1.42	0.80	2.31	NA	NA	NA
Combined treatment with remdesivir at any time	0.64	0.54	0.75	0.71	0.30	1.39
Combined treatment with remdesivir from day 1	0.54	0.39	0.74	NA	NA	NA

Samples sizes are listed in Table 1 (for severe COVID‐19) and Table 5 (for non‐hypoxic COVID‐19).

CI = 95% confidence interval; COVID‐19 = coronavirus disease 2019; NA = sample size too small, with complete separation of data; OR = odds ratio.

It was not possible to incorporate the time since the start of the pandemic into the propensity score, as it was an almost perfect predictor of treatment with dexamethasone or remdesivir, due to the approval of these drugs in June 2020. However, sensitivity analyses excluding cases prior to the approval of remdesivir and dexamethasone in the United Kingdom (which removed the collinearity between treatment and time) and including the time since the start of the pandemic as a covariate in the generation of the propensity score, yielded similar results, as shown in Figure 2B. Another sensitivity analysis was conducted excluding ventilation status from the calculation of the propensity score, because this variable is time‐dependent and ventilation may conceivably have been initiated before treatment. Results were similar in this unadjusted analysis (Fig 2C).

### Non‐Hypoxic COVID‐19

We assessed patients with non‐hypoxic COVID‐19 for two reasons. First, neurological complications of COVID‐19 may occur in the absence of severe respiratory disease.^37^ Second, we were also interested to assess whether remdesivir and/or dexamethasone prevented neurological complications in patients with milder respiratory disease. Patients with non‐hypoxic COVID‐19 (n = 25,209) had a median age of 72 years (IQR = 54–83 years). Fifty percent were men and 72% were White (see Table 1 and Table S3 for further details). They had a lower frequency of neurological complications compared with patients with severe disease (4.5% vs 4.8%, *χ* ^2^ = 3.95, *p* = 0.047). Compared with patients with severe COVID‐19, ICU admission was less frequent (0.9% vs 21%), as was mortality (9.2% vs 32%), worse self‐care at discharge (13% vs 19%), and median time to recovery (5 days, IQR of 2–10 days vs 8 days, IQR of 5–14 days). Patients with non‐hypoxic COVID‐19 and neurological complications (n = 1,141), compared to patients with non‐hypoxic COVID‐19 without neurological complications (n = 24,068; Table S2), had increased mortality (OR = 1.49, 95% CI = 1.24–1.78), ICU admission (OR = 2.45, 95% CI = 1.45–3.89), likelihood of worse self‐care on discharge (OR = 3.42, 95% CI = 2.89–4.03), and time to recovery (5.41 days, 95% CI = 4.37–6.45 days). Treatment with dexamethasone (n = 2,580) was associated with less neurological complications (OR = 0.78, 95% CI = 0.62–0.97), whereas combined treatment with dexamethasone and remdesivir at any time (n = 460) trended toward an association with a lower frequency of neurological complications (OR = 0.63, 95% CI = 0.31–1.15). There was no clear association for patients receiving remdesivir at any time (OR = 1.29, 95% CI = 0.50–2.70) and there were insufficient non‐hypoxic patients treated with remdesivir on the day of admission for analysis. In an exploratory subgroup analysis, a significantly lower frequency of stroke was associated with dexamethasone (OR = 0.47, 95% CI = 0.29–0.73); otherwise, there were insufficient numbers for analysis, or no association was seen (see Table 4).

As these treatments were prescribed off‐label in non‐hypoxic patients, we assessed if specific organ system complications were associated with an increased likelihood of treatment. Chronic pulmonary disease, immunosuppression, and obesity were associated with an increased likelihood of treatment with dexamethasone, whereas chronic cardiac, hematological, renal, and neurological disease were associated with a reduced likelihood of treatment (Table 5). A sensitivity analysis was conducted replacing the comorbidity score with these individual comorbidities in the derivation of the propensity score; the result was similar, with dexamethasone being associated with a lower frequency of neurological complications (OR = 0.76, 95% CI = 0.62–0.93). There were insufficient non‐hypoxic patients receiving remdesivir or combined treatment to draw conclusions about different frequencies of comorbidities in these groups.

**TABLE 5:** Association Between Individual Comorbidities and Dexamethasone Treatment in Patients with Non‐Hypoxic COVID‐19

Comorbidity	No dexamethasone	Dexamethasone	*χ* ^2^	*p* *
	n = 22,169	n = 3,040		
Chronic cardiac disease, n (%)	6,641 (30)	792 (26)	18.24	<0.001
Data missing	136	32		
Chronic hematological disease, n (%)	887 (4.0)	85 (2.8)	9.90	0.002
Data missing	138	32		
Chronic renal disease, n (%)	3,711 (17)	416 (14)	17.21	<0.001
Data missing	132	32		
Chronic rheumatological disease, n (%)	2,637 (12)	320 (11)	4.33	0.038
Data missing	138	34		
Chronic liver disease, n (%)	424 (1.9)	36 (1.2)	7.35	0.007
Data missing	135	34		
Chronic neurological disease, n (%)	5,184 (24)	528 (18)	53.26	<0.001
Data missing	139	33		
Immunosuppression, n (%)	2,103 (9.8)	355 (12)	16.60	<0.001
Data missing	727	144		
Obesity, n (%)	1,819 (8.3)	317 (11)	17.44	<0.001
Data missing	143	34		
Diabetes, n (%)	5,745 (26)	807 (27)	0.43	0.51
Data missing	317	37		
Chronic pulmonary disease, n (%)	3,292 (15)	537 (18)	19.65	<0.001
Data missing	642	137		
Malignancy, n (%)	2,349 (11)	279 (9.7)	4.00	0.046
Data missing	694	160		
Immunodeficiency, n (%)	90 (0.4)	11 (0.4)	0.02	0.89
Data missing	723	158		
Transplant, n (%)	238 (1.1)	28 (1.0)	0.33	0.57
Data missing	723	160		
Hypertension, n (%)	9,446 (43)	1,282 (43)	0.29	0.59
Data missing	312	36		

Further detail on each comorbidity is available in the predefined protocol.^26^ All percentages are that of the group represented in the column.

* Pearson's *χ* ^2^ test. To account for multiple comparisons *p* values <0.0036 were considered significant (Bonferroni).

COVID‐19 = coronavirus disease 2019.

## Discussion

### Neurological Complications

This is the first treatment study in COVID‐19 to focus on neurological complications. We show that patients with neurological complications related to COVID‐19 have significantly increased mortality and morbidity, in agreement with published data.^1^, ^6^ Treatment with either remdesivir or dexamethasone was associated with a significant reduction in the frequency of neurological complications following severe COVID‐19. Moreover, an even greater reduction was observed in patients receiving both drugs, which has important clinical implications. Our findings support the continued use of both dexamethasone and remdesivir in severe COVID‐19, and give reassurance that the survival advantage conferred by treatment with dexamethasone is not accompanied by an increase in neurological complications in survivors. The effect of remdesivir treatment alone on neurological complications, as well as the novel finding of an additive effect of the dexamethasone and remdesivir combination, encourages further detailed analysis into the benefits of remdesivir and combination therapies in COVID‐19.^38^

The lower frequency of neurological complications with treatment is unlikely to be due to patients with neurological complications having less severe respiratory disease, and therefore not requiring dexamethasone or remdesivir, for several reasons. First, the whole ethos of the propensity score is to control for the propensity of patients receiving the treatment, and a requirement for ventilation was used to control for respiratory disease severity within the propensity score. In addition, within the population with severe COVID‐19, ventilation (and therefore severe respiratory disease) was significantly associated with neurological complications (*χ* ^2^ = 93.06, *p* = 2.2 × 10^−16^), rather than the reverse. Furthermore, we conducted separate analyses in hypoxic (severe) and non‐hypoxic groups, and therefore hypoxia is highly unlikely to have been a confounder within these individual analyses. Finally, non‐hypoxic patients had a lower frequency of neurological complications, compared with patients with severe (hypoxic) COVID‐19 (4.5% vs 4.8%, *χ* ^2^ = 3.95, *p* = 0.047), rather than the reverse.

### Mortality and Morbidity

The effects of treatment with dexamethasone and remdesivir on mortality in patients with severe COVID‐19 were consistent with pivotal randomized controlled trials^2^, ^16^, ^29^ and previous studies of the ISARIC cohort,^24^ validating our analysis. Combined treatment with remdesivir and dexamethasone was associated with a reduction in both mortality and the likelihood of worse self‐care to a greater extent than dexamethasone alone in patients with severe COVID‐19, potentially demonstrating a synergistic effect not previously reported. The increased ICU admission seen in patients treated with remdesivir is likely to reflect the increased likelihood of its prescription to the sickest patients requiring higher levels of care.

### Non‐Hypoxic COVID‐19

Relative to some studies,^2^, ^18^ mortality was high (32%) among patients with severe COVID‐19. This was likely to be a fair representation for the early phase of the pandemic in the United Kingdom: CCP‐UK enrollment rates were high because recruitment did not require consent, and the cohort was estimated to represent two thirds of all admissions in this period. Nonetheless, this finding underlined the importance of performing the predefined separate analysis in patients who were non‐hypoxic throughout their admission. In this group, dexamethasone treatment was also associated with a lower frequency of neurological complications, whereas combined treatment with dexamethasone and remdesivir trended toward a lower frequency. It should be noted that, in these cases, treatment with dexamethasone or remdesivir would have been given outside national treatment guidelines, raising the possibility of selection bias by treating clinicians. For instance, we found evidence that chronic pulmonary conditions, immunosuppression, and obesity were associated with an increased likelihood of treatment with dexamethasone, but a sensitivity analysis correcting for these yielded similar results to the primary analysis. It also remains possible that patients with non‐hypoxic COVID‐19 had severe disease affecting organs outside the respiratory system.

Recent guidelines are starting to recommend treatment in selected non‐hospitalized vulnerable patients with non‐hypoxic COVID‐19.^33^ Our findings encourage future work to assess whether dexamethasone and remdesivir may prevent neurological complications in such patient groups, or if they impact on the changes in brain structure^9^ and cognition^39^ that have been identified in similar patients.

### Strengths and Limitations

Prior to applying eligibility criteria, the CCP‐UK study cohort included around two thirds of people hospitalized with COVID‐19 across the United Kingdom during the initial state of the pandemic. This large sample of real‐life data strengthens the generalizability of our findings and permitted an analysis of both single and combined treatment with remdesivir and dexamethasone. The use of the same control population throughout allowed for valid comparisons between groups. Finally, this is the first COVID‐19 treatment study focusing on neurological complications and their associated morbidity. The dataset showed conformity with published data; for instance, the circa 5% frequency of major neurological complications is close to that of an Italian study,^40^ a significantly lower frequency of major neurological complications was seen with milder respiratory COVID‐19 as has been noticed before,^41^ and a beneficial effect of dexamethasone on mortality was confirmed.^2^ Although there was an observed association between ventilation and neurological complications, this justifies its inclusion within the propensity score.

There are several important limitations to note. Many of these reflect the fact that participating clinicians and data collection staff were working under challenging conditions during the pandemic (where consecutive recruitment may been difficult to sustain in some centers), and that the CCP‐UK protocol and CRF, being agnostic with regard to pathogen and sufficiently pragmatic to allow rapid data collection, were not designed a priori to address the aims of our analysis plan. For instance, validated tools to measure long‐term functional outcomes were not used, and instead minimum outcome data was recorded on the 28th day of admission.

Because treatments were not randomized, associations between receiving treatment and the frequency of neurological complications should be interpreted with caution. To minimize this confounding, covariates were selected a priori to generate propensity scores reflecting the likelihood for patients to receive these treatments. The adequacy of the adjustment for confounders by the propensity score was validated by observing the different effects of dexamethasone and remdesivir on mortality previously reported from randomized controlled trials. However, one cannot definitively rule out additional confounding by covariates specific to neurological complications (rather than mortality) which were not included in the model. Hence, it is still possible that the patient's clinical state influenced the decision to use dexamethasone and/or remdesivir, and certainty can only be achieved with a randomized controlled trial.

Moreover, data were not collected on the timing of onset of neurological complications or dexamethasone treatment, so a time‐to‐event analysis was not possible. This was partially addressed by incorporating time from symptom onset to admission in propensity score calculation. It was not possible to incorporate the time since the start of the pandemic into the propensity score as it was an almost perfect predictor of treatment with dexamethasone or remdesivir and introduced significant skew into the propensity score distributions. This may have reduced the ability of the propensity scoring methodology to correct for confounding due to improved care over time for patients with COVID‐19. However, sensitivity analyses assessing treatment groups from the point dexamethasone was licensed in the United Kingdom (which removed the correlation between these treatment groups and time since the pandemic) and including the time since the start of the pandemic as a covariate in the generation of the propensity score, yielded similar results (see Fig 2B).

Finally, full vaccination against SARS‐CoV‐2 is becoming the norm in developed countries,^25^, ^26^, ^27^ but it is unclear whether and how vaccination affects the risk of developing related neurological complications when COVID‐19 occurs in a vaccinated population. Due to the constraints of our study period, there was insufficient power to assess the impact of treatments on neurological complications in vaccinated patients. For the same reason, emerging SARS CoV‐2 variants were not assessed and may respond differently to the analyzed treatments.

### Overall Conclusion and Further Directions

We have shown that neurological complications result in significant mortality and morbidity from COVID‐19, and a significantly lower frequency of these events was seen in association with dexamethasone and/or remdesivir treatment. This is of great importance to individual patients, health care systems, and public health bodies, although further studies are required to establish causality. A similar benefit in patients with non‐hypoxic COVID‐19 justifies future research to assess whether patients with less severe COVID‐19, who may still be at higher risk of neurological complications, are likely to benefit from these treatments. The potential additive and synergistic effects seen between remdesivir and dexamethasone, on neurological complications and mortality, respectively, warrant further assessment alongside similar analyses of other licensed COVID‐19 treatments, such as tocilizumab^42^ and baricitinib.^43^ Whereas a significant proportion of the world's population will remain unvaccinated for the foreseeable future, further studies should assess treatment strategies in vaccinated patients and with other variants of concern, including omicron, to determine the applicability of our findings to these patients.

## Author Contributions

S.P., B.D.M., R.H.T., and I.G. contributed to the conception and design of the study. A.G., C.H.W., M.H., K.B., P.J.M.O., M.G.S., D.B., and I.G. contributed to the acquisition and analysis of data. A.G., C.H.W., M.H., K.B., P.J.M.O., M.G.S., D.B., S.P., B.D.M., R.H.T., and I.G. contributed to drafting the text or preparing the figures.

## Potential Conflicts of Interest

I.G. was supported by Gilead Sciences (IN‐UK‐540‐6109), who market remdesivir, to conduct this work.

## Supporting information


**Appendix S1** Supporting information.

## Data Availability

ISARIC4C welcomes applications for data and material access through our Independent Data and Material Access Committee (https://isaric4c.net).

## References

[1] Drake TM , Riad AM , Fairfield CJ , et al. Characterisation of in‐hospital complications associated with COVID‐19 using the ISARIC WHO clinical characterisation protocol UK: a prospective, multicentre cohort study. Lancet 2021;398:223–237. 10.1016/S0140-6736(21)00799-6. 34274064 PMC8285118

[2] Group TRC . Dexamethasone in hospitalized patients with Covid‐19. 2020;384:693–704. 10.1056/NEJMOA2021436. PMC7383595 32678530

[3] Lechien JR , Chiesa‐Estomba CM , Place S , et al. Clinical and epidemiological characteristics of 1420 European patients with mild‐to‐moderate coronavirus disease. J Intern Med 2019;288:335–344. 10.1111/joim.13089 PMC7267446 32352202

[4] Consortium WST . Repurposed antiviral drugs for Covid‐19 —interim WHO solidarity trial results. 2020;384:497–511. 10.1056/NEJMOA2023184. PMC7727327 33264556

[5] Cagnazzo F , Arquizan C , Derraz I , et al. Neurological manifestations of patients infected with the SARS‐CoV‐2: a systematic review of the literature. J Neurol 2021;268:2656–2665. 10.1007/S00415-020-10285-9. 33125542 PMC7597753

[6] Meppiel E , Peiffer‐Smadja N , Maury A , et al. Neurologic manifestations associated with COVID‐19: a multicentre registry. Clin Microbiol Infect 2021;27:458–466. 10.1016/J.CMI.2020.11.005. 33189873 PMC7661948

[7] Varatharaj A , Thomas N , Ellul MA , et al. Neurological and neuropsychiatric complications of COVID‐19 in 153 patients: a UK‐wide surveillance study. Lancet Psychiatry 2020;7:875–882. 10.1016/S2215-0366(20)30287-X. 32593341 PMC7316461

[8] Patone M , Handunnetthi L , Saatci D , et al. Neurological complications after first dose of COVID‐19 vaccines and SARS‐CoV‐2 infection. Nat Med 2021;27:2144–2153. 10.1038/s41591-021-01556-7. 34697502 PMC8629105

[9] Douaud G , Lee S , Alfaro‐Almagro F , et al. SARS‐CoV‐2 is associated with changes in brain structure in UK Biobank. 10.1038/s41586-022-04569-5. PMC9046077 35255491

[10] Murray CJL , Piot P . The potential future of the COVID‐19 pandemic: will SARS‐CoV‐2 become a recurrent seasonal infection? JAMA 2021;325:1249–1250. 10.1001/JAMA.2021.2828. 33656519

[11] A T, A A, L C et al. After the pandemic: perspectives on the future trajectory of COVID‐19. Nature 2021;596:495–504. 10.1038/S41586-021-03792-W. 34237771

[12] Paterson RW , Brown RL , Benjamin L , et al. The emerging spectrum of COVID‐19 neurology: clinical, radiological and laboratory findings. Brain 2020;143:3104–3120. 10.1093/BRAIN/AWAA240. 32637987 PMC7454352

[13] Francistiová L , Klepe A , Curley G , et al. Cellular and molecular effects of SARS‐CoV‐2 linking lung infection to the brain. Front Immunol 2021;12. 10.3389/FIMMU.2021.730088. PMC8414801 34484241

[14] Pujadas E , Chaudhry F , McBride R , et al. SARS‐CoV‐2 viral load predicts COVID‐19 mortality. Lancet Respir Med 2020;8:e70. 10.1016/S2213-2600(20)30354-4. 32771081 PMC7836878

[15] Kaklamanos A , Belogiannis K , Skendros P , et al. COVID‐19 immunobiology: lessons learned, new questions Arise. Front Immunol 2021;12. 10.3389/FIMMU.2021.719023. PMC8427766 34512643

[16] Gupta M , Weaver DF . COVID‐19 as a trigger of brain autoimmunity. ACS Chem Nerosci 2021;12:2558–2561. 10.1021/ACSCHEMNEURO.1C00403. 34213312

[17] Kokic G , Hillen HS , Tegunov D , et al. Mechanism of SARS‐CoV‐2 polymerase stalling by remdesivir. Nat Commun 2021;12. 10.1038/S41467-020-20542-0. PMC7804290 33436624

[18] Beigel JH , Tomashek KM , Dodd LE , et al. Remdesivir for the treatment of Covid‐19—final report. 2020;383:1813–1826. 10.1056/NEJMOA2007764. PMC7262788 32445440

[19] Gottlieb RL , Vaca CE , Paredes R , et al. Early remdesivir to prevent progression to severe Covid‐19 in outpatients. N Engl J Med 2022;386:305–315. 10.1056/NEJMOA2116846. 34937145 PMC8757570

[20] Garibaldi BT , Wang K , Robinson ML , et al. Comparison of time to clinical improvement with vs without remdesivir treatment in hospitalized patients with COVID‐19. JAMA Netw Open 2021;4:e213071. 10.1001/JAMANETWORKOPEN.2021.3071. 33760094 PMC7991975

[21] Docherty AB , Harrison EM , Green CA , et al. Features of 20 133 UK patients in hospital with covid‐19 using the ISARIC WHO clinical characterisation protocol: prospective observational cohort study. BMJ 2020;369. 10.1136/BMJ.M1985. PMC7243036 32444460

[22] Yates T , Zaccardi F , Islam N , et al. Obesity, ethnicity, and risk of critical care, mechanical ventilation, and mortality in patients admitted to hospital with COVID‐19: analysis of the ISARIC CCP‐UK cohort. Obesity 2021;29:1223–1230. 10.1002/OBY.23178. 33755331 PMC8251439

[23] Drake TM , Fairfield CJ , Pius R , et al. Non‐steroidal anti‐inflammatory drug use and outcomes of COVID‐19 in the ISARIC clinical characterisation protocol UK cohort: a matched, prospective cohort study. Lancet Rheumatol 2021;3:e498–e506. 10.1016/S2665-9913(21)00104-1. 33997800 PMC8104907

[24] Arch B , Kovacs D , Scott J , et al. Evaluation of the effectiveness of remdesivir in treating severe COVID‐19 using data from the ISARIC WHO clinical characterisation protocol UK: a prospective, national cohort study. medRxiv 2021. 10.1101/2021.06.18.21259072.

[25] ISARIC4C consortium . Available at: https://isaric4c.net/. Accessed September 20, 2021.

[26] Grundmann A , Hardwick M , Wu C‐H , et al. Prevention of neurological complications during COVID‐19: Protocol for a retrospective analysis of the ISARIC4C national cohort. SSRN. Available at: https://papers.ssrn.com/sol3/papers.cfm?abstract_id=4037376. Accessed February 25, 2022.

[27] von Elm E , Altman DG , Egger M , et al. Strengthening the reporting of observational studies in epidemiology (STROBE) statement: guidelines for reporting observational studies. BMJ 2007;335:806–808. 10.1136/BMJ.39335.541782.AD. 17947786 PMC2034723

[28] Ross Russell AL , Hardwick M , Jeyanantham A , et al. Spectrum, risk factors and outcomes of neurological and psychiatric complications of COVID‐19: a UK‐wide cross‐sectional surveillance study. Brain Commun 2021;3. 10.1093/BRAINCOMMS/FCAB168. PMC8364668 34409289

[29] Rockwood K , Song X , MacKnight C , et al. A global clinical measure of fitness and frailty in elderly people. CMAJ 2005;173:489–495. 10.1503/CMAJ.050051. 16129869 PMC1188185

[30] CHMP . Annex I summary of product characteristics. https://www.medicines.org.uk/emc/product/11597/smpc, Accessed October 30, 2022.

[31] CAS‐ViewAlert . Available at: https://www.cas.mhra.gov.uk/ViewandAcknowledgment/ViewAlert.aspx?AlertID=103054. Accessed March 1, 2022.

[32] Austin PC , Mamdani MM . A comparison of propensity score methods: a case‐study estimating the effectiveness of post‐AMI statin use. Stat Med 2006;25:2084–2106. 10.1002/SIM.2328. 16220490

[33] RStudio Team . RStudio 2022 (Published online).

[34] Ho DE , Imai K , King G , Stuart EA . MatchIt: nonparametric preprocessing for parametric causal inference. J Stat Softw 2011;42:1–28. 10.18637/JSS.V042.I08.

[35] Rochwerg B , Agarwal A , Zeng L , et al. Remdesivir for severe covid‐19: a clinical practice guideline. BMJ 2020;370. 10.1136/BMJ.M2924. 32732352

[36] Ma S , Xu C , Liu S , et al. Efficacy and safety of systematic corticosteroids among severe COVID‐19 patients: a systematic review and meta‐analysis of randomized controlled trials. Signal Transduct Target Ther 2021;6:1–7. 10.1038/s41392-021-00521-7. 33612824 PMC7897363

[37] Pinto AA , Carroll LS , Nar V , et al. CNS inflammatory vasculopathy with antimyelin oligodendrocyte glycoprotein antibodies in COVID‐19. Neurolo Neuroimmunol Neuroinflamm 2020;7. 10.1212/NXI.0000000000000813. PMC7309522 32522768

[38] Gavriatopoulou M , Ntanasis‐Stathopoulos I , Korompoki E , et al. Emerging treatment strategies for COVID‐19 infection. Clin Exp Med 2021;21:167. 10.1007/S10238-020-00671-Y. 33128197 PMC7598940

[39] Liu YH , Chen Y , Wang QH , et al. One‐year trajectory of cognitive changes in older survivors of COVID‐19 in Wuhan, China: a longitudinal cohort study. JAMA Neurol 2022;79:509–517. 10.1001/JAMANEUROL.2022.0461. 35258587 PMC8905512

[40] Rifino N , Censori B , Agazzi E , et al. Neurologic manifestations in 1760 COVID‐19 patients admitted to papa Giovanni XXIII hospital, Bergamo, Italy. J Neurol 2021;268:2331. 10.1007/S00415-020-10251-5. 33026520 PMC7539268

[41] Liotta EM , Batra A , Clark JR , et al. Frequent neurologic manifestations and encephalopathy‐associated morbidity in Covid‐19 patients. Ann Clin Transl Neurol 2020;7:2221–2230. 10.1002/ACN3.51210. 33016619 PMC7664279

[42] Gupta S , Wang W , Hayek SS , et al. Association between early treatment with Tocilizumab and mortality among critically ill patients with COVID‐19. JAMA Intern Med 2021;181:41–51. 10.1001/JAMAINTERNMED.2020.6252. 33080002 PMC7577201

[43] Marconi VC , Ramanan AV , de Bono S , et al. Efficacy and safety of baricitinib for the treatment of hospitalised adults with COVID‐19 (COV‐BARRIER): a randomised, double‐blind, parallel‐group, placebo‐controlled phase 3 trial. Lancet Respir Med 2021;9:1407–1418. 10.1016/S2213-2600(21)00331-3. 34480861 PMC8409066

