# Supplementary material for: Fewer COVID‐19 Neurological Complications with Dexamethasone and Remdesivir
Source: Ann Neurol. 2022 Nov 9;93(1):88–102. doi: 10.1002/ana.26536 (PMC9874556; doi:10.1002/ana.26536)
Supplement: Supplementary file 1 — Appendix S1 Supporting information. [file ANA-93-88-s001.pdf]

Supporting Information for:

Fewer COVID-19 neurological complications with dexamethasone and remdesivir

Dr Alexander Grundmann MD[1,2*], Dr Chieh-Hsi Wu PhD[3*] Dr Marc Hardwick MD[1,2*], Prof J Kenneth Baillie MD PhD[4,5], Prof Peter JM Openshaw MD PhD[6,7], Prof Malcolm G Semple MD PhD[8,9], Prof Dankmar Böhning PhD[3], Prof Sarah Pett MD PhD[10,11], Dr Benedict D Michael MD PhD[8,12,13#], Dr Rhys H Thomas MD PhD[14,15#], Prof Ian Galea MD PhD[1,2#] for ISARIC4C investigators.

1. Clinical Neurosciences, Clinical and Experimental Sciences, Faculty of Medicine, University of Southampton, UK
2. Department of Neurology, Wessex Neurological Centre, University Hospital Southampton NHS Foundation Trust, UK
3. Statistics, Mathematical Sciences, Faculty of Social Sciences, University of Southampton, UK
4. Roslin Institute, University of Edinburgh, Easter Bush, Edinburgh, UK
5. Intensive Care Unit, Royal Infirmary of Edinburgh, Edinburgh, UK.
6. National Heart and Lung Institute, Imperial College London, London, UK
7. Imperial College Healthcare NHS Trust: London, London, UK
8. NIHR Health Protection Research Unit for Emerging and Zoonotic Infections, Institute of Infection, Veterinary and Ecological Sciences, University of Liverpool, Liverpool, UK;
9. Department of Respiratory Medicine, Alder Hey Children's Hospital, Liverpool, UK
10. Medical Research Council Clinical Trials Unit, Institute of Clinical Trials and Methodology, University College London, UK
11. Institute for Global Health, University College London, UK
12. Department of Clinical Infection Microbiology and Immunology, Institute of Infection, Veterinary, and Ecological Sciences, University of Liverpool, UK
13. Department of Neurology, The Walton Centre NHS Foundation Trust, UK
14. Translational and Clinical Research Institute, University of Newcastle, UK
15. Department of Neurology, Royal Victoria Infirmary, UK

* Joint primary authors
# Joint senior authors

Characteristic	Overall n = 64,088	No treatment n = 30,685	Dexamethasone n = 21,129	Remdesivir at any time n = 1,428	Remdesivir from day 1 n = 354	Combined treatment with remdesivir at any time n = 10,846	Combined treatment with remdesivir from day 1 n = 2,737
**Age, median (IQR)**	70 (56 – 81)	74 (60 – 84)	68 (55 – 79)	63 (53 – 74)	60 (51 – 71)	62 (52 – 74)	60 (50 – 70)
**Sex, n (%)**							
Female	26,460 (41)	13,180 (43)	8,662 (41)	535 (37)	126 (36)	4,083 (38)	975 (36)
Male	37,544 (59)	17,465 (57)	12,435 (59)	891 (62)	228 (64)	6,753 (62)	1,761 (64)
Not specified	68 (0.1)	30 (<0.1)	28 (0.1)	2 (0.1)	0 (0)	8 (<0.1)	1 (<0.1)
Data missing	16	10	4	0	0	2	0
**Ethnicity, n (%)**							
BAME	6,787 (11)	2,923 (9.6)	2,263 (11)	204 (14)	49 (14)	1,397 (13)	377 (14)
White	45,499 (72)	22,335 (73)	15,148 (72)	923 (65)	213 (61)	7,093 (66)	1,674 (62)
Other	3,799 (6.0)	1,681 (5.5)	1,259 (6.0)	89 (6.3)	24 (6.9)	770 (7.2)	207 (7.6)
Not specified	7,491 (12)	3,549 (12)	2,266 (11)	197 (14)	64 (18)	1,479 (14)	451 (17)
Data missing	512	197	193	15	4	107	28
**Comorbidities score, median (IQR)**	2.00 (1.00 – 3.00)	2.00 (1.00 – 3.00)	2.00 (1.00 – 3.00)	2.00 (1.00 – 3.00)	2.00 (1.00 – 3.00)	2.00 (1.00 – 3.00)	1.00 (0.00 – 2.00)
**Clinical Frailty Score, median (IQR)**	4.00 (2.00 – 6.00)	5.00 (3.00 – 7.00)	3.00 (2.00 – 5.00)	3.00 (2.00 – 5.00)	2.00 (2.00 – 4.00)	3.00 (2.00 – 4.00)	2.00 (2.00 – 3.00)
Data missing	28,827	13,303	9,534	783	197	5,207	1,376
**Smoking status, n (%)**							
Current smoker	2,851 (4.5)	1,474 (4.8)	874 (4.2)	55 (3.9)	9 (2.6)	448 (4.2)	101 (3.8)
Former smoker	14,931 (23)	6,834 (22)	5,221 (25)	316 (22)	62 (18)	2,560 (24)	588 (22)
Never smoked	18,782 (30)	8,080 (26)	6,390 (31)	413 (29)	118 (34)	3,899 (36)	1,026 (38)
Not specified	26,996 (42)	14,159 (46)	8,391 (40)	635 (45)	162 (46)	3,811 (36)	976 (36)
Data missing	528	138	253	9	3	128	46
**Ventilated, n (%)**	19,204 (30)	7,541 (25)	6,137 (29)	760 (53)	198 (56)	4,766 (44)	1,215 (44)
Data missing	57	38	11	0	0	8	2
**Time from symptom onset to admission, days, median (IQR)**	5.0 (2.0 – 9.0)	4.0 (1.0 – 8.0)	6.0 (2.0 – 9.0)	6.0 (3.0 – 8.0)	7.0 (4.0 – 9.0)	6.0 (3.0 – 9.0)	7.0 (4.0 – 9.0)
**Patients with neurological complication, n (%)**	3,104 (4.8)	1,967 (6.4)	780 (3.7)	56 (3.9)	18 (5.1)	301 (2.8)	63 (2.3)
**Seizure, n (%)**							
Not specified	2,595 (4.0)	1,495 (4.9)	785 (3.7)	51 (3.6)	11 (3.1)	264 (2.4)	51 (1.9)
Absent	60,799 (95)	28,652 (93)	20,240 (96)	1,372 (96)	343 (97)	10,535 (97)	2,675 (98)
Present	692 (1.1)	537 (1.8)	104 (0.5)	5 (0.4)	0 (0)	46 (0.4)	10 (0.4)
Data missing	2	1	0	0	0	1	1
**Stroke, n (%)**							
Not specified	2,626 (4.1)	1,533 (5.0)	789 (3.7)	51 (3.6)	10 (2.8)	253 (2.3)	49 (1.8)
Absent	60,539 (94)	28,506 (93)	20,159 (95)	1,363 (95)	340 (96)	10,511 (97)	2,670 (98)

Characteristic	**Overall** n = 64,088	**No treatment** n = 30,685	**Dexamethasone** n = 21,129	**Remdesivir at any time** n = 1,428	**Remdesivir from day 1** n = 354	**Combined treatment with remdesivir at any time** n = 10,846	**Combined treatment with remdesivir from day 1** n = 2,737
Present	923 (1.4)	646 (2.1)	181 (0.9)	14 (1.0)	4 (1.1)	82 (0.8)	18 (0.7)
**Meningitis/encephalitis, n (%)**							
Not specified	2,630 (4.1)	1,532 (5.0)	791 (3.7)	50 (3.5)	9 (2.5)	257 (2.4)	51 (1.9)
Absent	61,313 (96)	29,040 (95)	20,315 (96)	1,377 (96)	345 (97)	10,581 (98)	2,684 (98)
Present	142 (0.2)	112 (0.4)	22 (0.1)	1 (<0.1)	0 (0)	7 (<0.1)	1 (<0.1)
Data missing	3	1	1	0	0	1	1
**Other neurological complication, n (%)**							
Not specified	2,835 (4.4)	1,662 (5.5)	840 (4.0)	51 (3.6)	10 (2.8)	282 (2.6)	51 (1.9)
Absent	59,235 (93)	27,772 (92)	19,750 (94)	1,340 (94)	329 (93)	10,373 (96)	2,643 (97)
Present	1,642 (2.6)	884 (2.9)	530 (2.5)	37 (2.6)	15 (4.2)	191 (1.8)	43 (1.6)
Data missing	376	367	9	0	0	0	0
**Mortality, n (%)**	20,234 (32)	11,490 (37)	5,919 (28)	472 (33)	95 (27)	2,353 (22)	448 (16)
Data missing	4	2	0	0	0	2	0
**ICU admission, n (%)**	13,629 (21)	5,330 (17)	4,379 (21)	614 (43)	180 (51)	3,306 (31)	887 (32)
Data missing	62	37	15	1	0	9	1
**Worse self-care at discharge, n (%)**	7,778 (19)	3,590 (21)	2,582 (18)	220 (25)	56 (24)	1,386 (17)	343 (16)
Data missing	23,870	13,741	6,835	544	121	2,750	547
**Time to recovery, days, median (IQR)**	8 (5 – 14)	9 (5 – 16)	7 (4 – 12)	10 (6 – 16)	9 (5 – 15)	8 (6 – 13)	7 (5 – 11)
Data missing	25,841	14,513	7,540	625	139	3,163	616

Table S1. Demographic and clinical characteristics of patients with severe COVID-19, organised by treatment group. All percentages are that of the group represented in the column. Some patients had more than one complication so the sum of individual diagnoses is greater than the number of patients with neurological complications. IQR = interquartile range, ICU = intensive care unit

	Severe COVID-19		Non-hypoxic COVID-19	
**Characteristic**	**No neurological complication** n = 60,984	**Neurological complication** n = 3,104	**No neurological complication** n = 24,068	**Neurological complication** n = 1,141
**Age, median (IQR)**	70 (56 – 81)	73 (61 – 83)	72 (53 – 83)	78 (65 – 86)
**Sex, n (%)**				
Female	25,256 (41)	1,204 (39)	12,140 (50)	527 (46)
Male	35,647 (58)	1,897 (61)	11,895 (49)	613 (54)
Not specified	65 (0.1)	3 (<0.1)	29 (0.1)	1 (<0.1)
Data missing	16	0	4	0
**Ethnicity, n (%)**				
BAME	6,461 (11)	326 (11)	2,525 (11)	81 (7.2)
White	43,290 (72)	2,209 (72)	17,217 (72)	877 (78)
Other	3,609 (6.0)	190 (6.2)	1,287 (5.4)	40 (3.5)
Not specified	7,138 (12)	353 (11)	2,830 (12)	131 (12)
Data missing	486	26	209	12
**Comorbidities score, median (IQR)**	2.00 (1.00 – 3.00)	2.00 (1.00 – 3.00)	2.00 (1.00 – 3.00)	2.00 (1.00 – 3.00)
**Clinical Frailty Score, median (IQR)**	4.00 (2.00 – 6.00)	5.00 (3.00 – 7.00)	4.00 (2.00 – 6.00)	6.00 (4.00 – 7.00)
Data missing	27,350	1,477	11,765	566
**Smoking status, n (%)**				
Current smoker	2,671 (4.4)	180 (5.9)	1,414 (5.9)	77 (6.8)
Former smoker	14,283 (24)	648 (21)	4,315 (18)	217 (19)
Never smoked	17,910 (30)	872 (29)	7,008 (29)	328 (29)
Not specified	25,640 (42)	1,356 (44)	11,137 (47)	505 (45)
Data missing	480	48	194	14
**Ventilated, n (%)**	18,033 (30)	1,171 (38)	0 (0)	0 (0)
Data missing	55	2	0	0
**Time from symptom onset to admission, days, median (IQR)**	5.0 (2.0 – 9.0)	3.0 (0.0 – 7.0)	3 (0 – 7)	1 (0 – 6)
**Treatment group, n (%)**				
Dexamethasone	20,349 (33)	780 (25)	2,494 (10)	86 (7.5)
Remdesivir at any time	1,372 (2.2)	56 (1.8)	124 (0.5)	6 (0.5)

	Severe COVID-19		Non-hypoxic COVID-19	
**Characteristic**	**No neurological complication** n = 60,984	**Neurological complication** n = 3,104	**No neurological complication** n = 24,068	**Neurological complication** n = 1,141
Remdesivir from day 1	336 (0.6)	18 (0.6)	26 (0.1)	0 (0)
Combined treatment with remdesivir at any time	10,545 (17)	301 (9.7)	450 (1.9)	10 (0.9)
Combined treatment with remdesivir from day 1	2,674 (4.4)	63 (2.0)	117 (0.5)	1 (<0.1)
No treatment	28,718 (47)	1,967 (63)	21,000 (87)	1,039 (91)
**Mortality, n (%)**	18,832 (31)	1,402 (45)	2,123 (8.8)	184 (16)
Data missing	4	0	2	0
**ICU admission, n (%)**	12,652 (21)	977 (32)	215 (0.9)	20 (1.8)
Data missing	59	3	29	2
**Worse self-care at discharge, n (%)**	7,084 (18)	694 (50)	2,424 (12)	282 (35)
Data missing	22,153	1,717	3,663	337
**Time to recovery, d, median (IQR)**	8 (5 – 14)	18 (10 – 29)	5 (2 – 10)	10 (5 – 20)
Data missing	23,880	1,961	4,136	402

Table S2. Demographic and clinical characteristics of patients with severe and non-hypoxic COVID-19, organised by whether a neurological complication occurred. All percentages are that of the group represented in the column. IQR = interquartile range, ICU = intensive care unit, NA = not available

Characteristic	Overall<br>n = 25,209	No treatment<br>n = 22,039	Dexamethasone<br>n = 2,580	Remdesivir<br>at any time<br>n = 130	Remdesivir<br>from day 1<br>n = 26	Combined treatment with remdesivir at any time<br>n = 460	Combined treatment with remdesivir from day 1<br>n = 118
**Age, median (IQR)**	72 (54 – 83)	73 (54 – 84)	71 (54 – 82)	64 (53 – 77)	62 (50 – 74)	60 (48 – 73)	58 (45 – 67)
**Sex, n (%)**							
Female	12,667 (50)	11,268 (51)	1,146 (44)	55 (42)	12 (46)	198 (43)	49 (42)
Male	12,508 (50)	10,742 (49)	1,430 (55)	75 (58)	14 (54)	261 (57)	68 (58)
Not specified	30 (0.1)	25 (0.1)	4 (0.2)	0 (0)	0 (0)	1 (0.2)	1 (0.8)
Data missing	4	4	0	0	0	0	0
**Ethnicity, n (%)**							
BAME	2,606 (10)	2,224 (10)	295 (12)	18 (14)	4 (15)	69 (16)	23 (20)
White	18,094 (72)	16,030 (73)	1,727 (67)	79 (61)	13 (50)	258 (59)	63 (55)
Other	1,327 (5.3)	1,128 (5.2)	160 (6.3)	9 (7.0)	4 (15)	30 (6.8)	10 (8.7)
Not specified	2,961 (12)	2,474 (11)	380 (15)	23 (18)	5 (19)	84 (19)	19 (17)
Data missing	221	183	18	1	0	19	3
**Comorbidities score, Median (IQR)**	2.00 (1.00 – 3.00)	2.00 (1.00 – 3.00)	2.00 (1.00 – 3.00)	1.00 (1.00 – 2.00)	1.00 (1.00 – 2.00)	1.00 (0.00 – 2.00)	1.00 (0.00 – 2.00)
**Clinical Frailty Score, Median (IQR)**	4.00 (3.00 – 6.00)	4.00 (3.00 – 6.00)	4.00 (2.00 – 6.00)	3.00 (2.00 – 6.00)	2.50 (1.75 – 3.50)	3.00 (2.00 – 4.00)	2.00 (1.00 – 4.00)
Data missing	12,331	10,661	1,292	92	18	286	70
**Smoking status, n (%)**							
Current smoker	1,491 (6.0)	1,321 (6.0)	141 (5.5)	5 (3.9)	1 (3.8)	24 (5.3)	7 (6.0)
Former smoker	4,532 (18)	3,970 (18)	469 (18)	14 (11)	2 (7.7)	79 (17)	14 (12)
Never smoked	7,336 (29)	6,427 (29)	694 (27)	38 (29)	9 (35)	177 (39)	48 (41)
Not specified	11,642 (47)	10,149 (46)	1,245 (49)	72 (56)	14 (54)	176 (39)	48 (41)
Data missing	208	172	31	1	0	4	1
**Ventilated, n (%)**	0 (0)	0 (0)	0 (0)	0 (0)	0 (0)	0 (0)	0 (0)
**Time from symptom onset to admission, days, median (IQR)**	3 (0 – 7)	3 (0 – 7)	5 (1 – 9)	5 (2 – 8)	6 (2 – 9)	6 (3 – 8)	7 (4 – 9)
**Patients with neurological complication, n (%)**	1,141 (4.5)	1,039 (4.7)	86 (3.3)	6 (4.6)	0 (0)	10 (2.2)	1 (0.8)
**Seizure, n (%)**							
Not specified	1,184 (4.7)	1,057 (4.8)	116 (4.5)	6 (4.6)	2 (7.7)	5 (1.1)	1 (0.8)
Absent	23,792 (94)	20,769 (94)	2,447 (95)	123 (95)	24 (92)	453 (98)	117 (99)
Present	233 (0.9)	213 (1.0)	17 (0.7)	1 (0.8)	0 (0)	2 (0.4)	0 (0)
**Stroke, n (%)**							
Not specified	1,188 (4.7)	1,062 (4.8)	115 (4.5)	6 (4.6)	2 (7.7)	5 (1.1)	1 (0.8)
Absent	23,685 (94)	20,659 (94)	2,451 (95)	123 (95)	24 (92)	452 (98)	116 (98)
Present	336 (1.3)	318 (1.4)	14 (0.5)	1 (0.8)	0 (0)	3 (0.7)	1 (0.8)
**Meningitis/encephalitis, n (%)**							

**Characteristic**	**Overall**<br>n = 25,209	**No treatment**<br>n = 22,039	**Dexamethasone**<br>n = 2,580	**Remdesivir at any time**<br>n = 130	**Remdesivir from day 1**<br>n = 26	**Combined treatment with remdesivir at any time**<br>n = 460	**Combined treatment with remdesivir from day 1**<br>n = 118
Not specified	1,180 (4.7)	1,054 (4.8)	114 (4.4)	7 (5.4)	2 (7.7)	5 (1.1)	1 (0.8)
Absent	23,976 (95)	20,938 (95)	2,461 (95)	123 (95)	24 (92)	454 (99)	117 (99)
Present	52 (0.2)	46 (0.2)	5 (0.2)	0 (0)	0 (0)	1 (0.2)	0 (0)
Data missing	1	1	0	0	0	0	0
**Other neurological complication, n (%)**							
Not specified	1,284 (5.1)	1,141 (5.2)	128 (5.0)	6 (4.6)	2 (7.7)	9 (2.0)	2 (1.7)
Absent	23,220 (93)	20,259 (92)	2,395 (93)	120 (92)	24 (92)	446 (97)	116 (98)
Present	585 (2.3)	521 (2.4)	55 (2.1)	4 (3.1)	0 (0)	5 (1.1)	0 (0)
Data missing	120	118	2	0	0	0	0
**Mortality, n (%)**	2,307 (9.2)	1,944 (8.8)	304 (12)	10 (7.7)	1 (3.8)	49 (11)	14 (12)
Data missing	2	2	0	0	0	0	0
**ICU admission, n (%)**	235 (0.9)	197 (0.9)	21 (0.8)	10 (7.7)	1 (3.8)	7 (1.5)	2 (1.7)
Data missing	31	26	3	0	0	2	0
**Worse self-care at discharge, n (%)**	2,706 (13)	2,412 (13)	261 (12)	12 (11)	0 (0)	21 (5.4)	5 (5.1)
Data missing	4,000	3,491	415	22	1	72	19
**Time to recovery, days, median (IQR)**	5 (2 – 10)	5 (2 – 10)	5 (2 – 10)	7 (4 – 10)	4 (3 – 7)	6 (4 – 10)	4 (3 – 8)
Data missing	4,538	3,980	453	24	1	81	20

Table S3. Demographic and clinical characteristics of patients with non-hypoxic COVID-19, organized by treatment group. All percentages are that of the group represented in the column. Some patients had more than one complication so the sum of individual diagnoses is greater than the number of patients with neurological complications. IQR = interquartile range, ICU = intensive care unit
